# Impact of trichostatin A and sodium valproate treatment on post-stroke neurogenesis and behavioral outcomes in immature mice

**DOI:** 10.3389/fncel.2013.00123

**Published:** 2013-08-19

**Authors:** Shanu George, Shilpa D. Kadam, Natasha D. Irving, Geoffrey J. Markowitz, Saba Raja, Anthony Kwan, YuShan Tu, Huigen Chen, Charles Rohde, Dani R. Smith, Anne M. Comi

**Affiliations:** ^1^Department of Neurology and Developmental Medicine, Hugo Moser Kennedy Krieger Research InstituteBaltimore, MD, USA; ^2^Neuroscience, Kennedy Krieger InstituteBaltimore, MD, USA; ^3^Department of Neurology, Johns Hopkins School of MedicineBaltimore, MD, USA; ^4^Department of Biostatistics, Johns Hopkins School of Public HealthBaltimore, MD, USA; ^5^Neurogenetics and Behavior Center, Department of Psychological and Brain Sciences, Johns Hopkins UniversityBaltimore, MD, USA; ^6^Department of Pediatrics, Johns Hopkins School of MedicineBaltimore, MD, USA

**Keywords:** valproate, trichostatin A, neonatal stroke, hippocampal neurogenesis, behavioral outcomes, anticonvulsants, histone deacetylase inhibitors

## Abstract

Stroke in the neonatal brain frequently results in neurologic impairments including cognitive disability. We investigated the effect of long-term sodium valproate (valproate) and trichostatin A (TSA) treatment upon post-stroke neurogenesis in the dentate gyrus (DG) of stroke-injured immature mice. Decreased or abnormal integration of newborn DG neurons into hippocampal circuits can result in impaired visual-spatial function, abnormal modulation of mood-related behaviors, and the development of post-stroke epilepsy. Unilateral carotid ligation of P12 CD1 mice was followed by treatment with valproate, TSA, or vehicle for 2 weeks, bromodeoxyuridine (BrdU) administration for measurement of neurogenesis, and perfusion at P42 or P60. Behavior testing was conducted from P38–42. No detrimental effects on behavior testing were noted with TSA treatment, but mildly impaired cognitive function was noted with valproate-treated injured animals compared to normal animals. Significant increases in DG neurogenesis with both TSA and valproate treatment were noted with later administration of BrdU. Increased mortality and impaired weight gain was noted in the valproate-treated ligated animals, but not in the TSA-treated animals. In summary, the impact of histone deacetylase (HDAC) inhibition upon post-stroke subgranular zone neurogenesis is likely to depend on the age of the animal at the time point when neurogenesis is assessed, duration of HDAC inhibition before BrdU labeling, and/or the stage in the evolution of the injury.

## INTRODUCTION

Neonatal stroke commonly results in cognitive impairments and other neurologic consequences in approximately 75% of survivors. While the extent of functional recovery after neonatal stroke can be remarkable due to the ongoing neuroplasticity and brain development, nevertheless the lifetime consequences of the neonatal stroke remain significant. A fundamental question of interest is how the enhanced neuroplasticity of the immature brain can best be harnessed to improve long-term outcomes. One class of drugs with potential to positively influence post-stroke neurogenesis and regeneration is histone deacetylase (HDAC) inhibitors. Recent studies have provided significant evidence of neuroprotection by HDAC inhibitors after stroke in the mature brain associated with HDAC4 acetylation (Liu et al., 2012), increased angiogenesis via up-regulation of Hif-1α (Wang et al., 2012), decreased inflammatory markers and activated microglia, HSP70 induction, increased phospho-Akt, and decreased pro-apoptotic proteins such as p53 (Kim et al., 2007). The few available studies suggest evidence of neuroprotection in the immature brain as well (Kabakus et al., 2005; Sandner et al., 2011; Fleiss et al., 2012). Studies addressing the impact of HDAC inhibitors upon post-stroke neurogenesis remain extremely limited (Kim et al., 2009; Liu et al., 2012), however, particularly in the immature brain. Many aspects of the evolving post-stroke injury and regenerative response differ in the immature brain from that in the mature brain, and therefore studies should be done in clinically relevant animal models of neonatal stroke.

Histone deacetylases, of which there are currently 11 in 5 different sub-groups, are enzymes involved in the epigenetic regulation of gene expression and protein function, and are partly responsible for the control of long-term changes in neuronal functions. Histone acetyltransferase (HAT) facilitates histone acetylation, releases condensed chromatin, and increases gene transcription (Bannister and Kouzarides, 2011). HDACs, on the other hand, remove acetyl groups from histone, thereby promoting chromatin condensation and repression of gene transcription. HDAC inhibitors can, however, also decrease activation of some genes by increasing gene repressor transcription. Furthermore, non-histone proteins important in microtubule stability have also been shown to serve as substrates for certain HDACs (Zhang et al., 2003), demonstrating the importance of acetylation in post-translational protein regulation. Therefore their effects are expected to be complex and dependent upon the developmental and contextual milieu. Valproate has been shown to affect gene expression in millimolar concentrations through its direct action as an inhibitor of primarily class 1 HDACs (Gottlicher et al., 2001; Phiel et al., 2001), and to a lesser extent class 2, but not HDAC6 or HDAC10 (Gurvich et al., 2004) The hydroxamic acid trichostatin A ([*R*-(*E,E*)]-7-[4-(dimethylamino) phenyl]-*N*-hydroxy-4,6-dimethyl-7-oxo-2,4-heptadienamide; TSA) is a potent HDAC inhibitor, structurally dissimilar to valproate, demonstrated to inhibit class 1 and class 2 HDACs in nanomolar amounts (Yoshida and Horinouchi, 1999).

Both valproate and TSA have been shown to suppress cell growth by cell cycle arrest and promotion of cell differentiation (Drummond et al., 2005). This profile has led to their wide study in the setting of cancer. Effects of valproate on the stimulation of neurogenesis and neurotrophic pathways have also been reported. Valproate has been shown to promote neural differentiation via the increased expression of the proneural genes Ngn1, Math1, and P15 associated with the increased acetylation of H4 (Yu et al., 2009). Increased neurogenesis after ischemia in a rat model treated with sodium butyrate was associated with increased expression of BDNF and phospho-Creb (Kim et al., 2009). Developmental differences have been noted in the role of HDACs in neurogenesis. For example, there is evidence that HDAC2 is critical for adult neurogenesis, but not required for embryonic neurogenesis (Jawerka et al., 2010); therefore HDAC inhibitor selectivity is important as is the context of its use.

Recent studies suggest that HDAC inhibitors are also effective for treating neurodegenerative disorders or enhancing synaptic plasticity (Hockly et al., 2003; Vecsey et al., 2007). Disrupted cellular acetylation homeostasis of histones and other proteins has been shown to be a common feature in neuropathological states, including stroke (Chuang et al., 2009; Selvi et al., 2010). Neurodegeneration is associated with decreased HAT activity, resulting in relative over-deacetylation. HDAC inhibitors have therefore been tested for therapeutic efficacy with promising results in models of stroke, Huntington disease, amyotrophic lateral sclerosis and experimental autoimmune encephalomyelitis. Similarly, the number of acetylated histone (AH3) positive cells was decreased by cerebral ischemia in a stroke model and restored by HDAC inhibitor treatment in the ipsilateral dentate gyrus (DG) 14 days after the stroke injury. Most AH3 (+) cells were found to co-localize with neuronal nuclei (NeuN) marker indicating an association of HDAC inhibition with the increased neurogenesis (Kim et al., 2009). Very little is known about the effect of HDAC inhibitors in the immature brain, however, with or without an overriding developmental insult such as stroke. While valproate is not considered an anticonvulsant of choice for infants, because of the risk of liver failure, nevertheless studying its effects as an anticonvulsant with HDAC inhibitory effects may serve as a proof of principle from which to derive novel regenerative approaches.

Neurogenesis has been shown to occur in the brain at a high rate during embryonic and early postnatal development and to persist at a slower rate for the remainder of life. Although postnatal neurogenesis has been reported to exist in many brain areas, it is seen most consistently in two: the DG of the hippocampus and the subventricular zone of the lateral ventricles. The hippocampus has long been implicated in memory recall, learning, emotion, and cognition; with ablations or lesions usually leading to deficits in associated tasks (Scoville and Milner, 1957; Kim et al., 2012). In the DG, cells added later in life are similar in connectivity and firing pattern as those born during embryonic development (Laplagne et al., 2007). Studies have been conducted showing immediate early gene upregulation in newborn neurons following novel exploration experiences (Kadam et al., 2010). Together, this indicates that these newborn cells have the capability of functionally integrating with existing circuits. The precise effect of altered levels of neurogenesis on cognitive performance remains an active subject of research.

The unilateral carotid ligation model used here with double ligation of the right common carotid ligation, is a modification of the original carotid ligation model (Comi et al., 2004), and produces a range of injury to the cortex, hippocampus, thalamus and striatum as described in this initial manuscript. We have found that this type of insult produces stroke and injury with more than 70% of the ligated P12 CD1 mice (Comi et al., 2004). The double carotid ligation is done to ensure ligation of the vessel but does not alter the extent of the injury. The carotid ligation P12 mouse model of stroke in the immature brain has been associated with decreased subgranular zone (SGZ) proliferation and neurogenesis (Kadam et al., 2008, 2009a) and impairments in cognitive function related to hippocampal function (Kadam et al., 2009b). One advantage of the P12 mouse carotid ligation model is that it induces acute behavioral seizures; this feature mimics the presentation of neonatal stroke with seizures in human infants and, because the severity of the acute seizures correlates with the severity of the brain injury, we use this feature to distribute the injured animals between the drug and vehicle treatment groups.

The goal of this study was to determine the long-term effects of an anticonvulsant known to be an HDAC inhibitor, and that of a potent HDAC inhibitor with no known anticonvulsant action (Hoffmann et al., 2008), on post-stroke neurogenesis and behavioral outcome. We therefore tested valproate and TSA in a CD1 mouse model of stroke in the immature brain. We hypothesized that both HDAC inhibitors would promote post-stroke neurogenesis in the immature brain.

## MATERIALS AND METHODS

**FIGURE 1:**
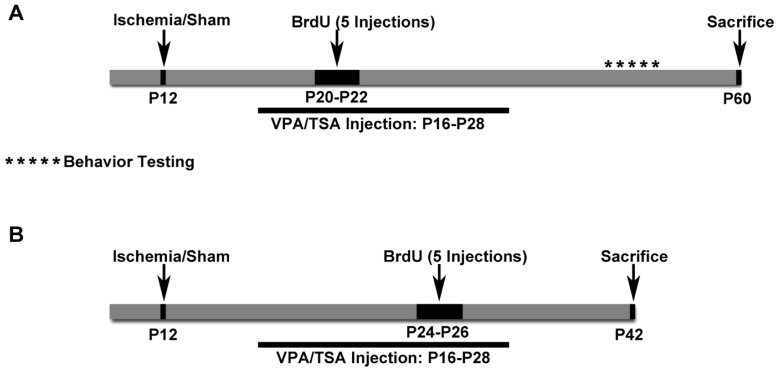
**Schematics of Protocols 1 and 2. (A)** Schematic for Protocol 1. Cognitive testing was done with these animals. **(B)** Schematic for Protocol 2. These mice received BrdU later and were euthanized sooner than in Protocol 1. These mice did not have cognitive testing done.

All materials and methods were approved by the Johns Hopkins University Animal Care and Use Committee.**Litters of CD1 mice were purchased from Charles River Laboratories Inc. (Wilmington, MA, USA). Pups were housed in polycarbonate cages with the dam on a 12-h light:dark cycle; food was provided *ad libitum*. Pups from 13 litters used for Protocol 1 (see **Table 1** for details) were randomly assigned to one of the four treatment groups [i.e., 200 mg/kg valproate twice a day, a dose selected as both anticonvulsant in rodents and effective as an HDAC inhibitor (Hoffmann et al., 2008), saline, 2.5 mg/kg TSA twice a day, or 5% DMSO] beginning 4 days after ligation. For Protocol 2, a total of three litters of animals were used for testing TSA versus 5% DMSO control, five litters were used for testing valproate versus saline control, and pups were randomly assigned to receive drug versus saline control except that animals with seizures were evenly distributed between the two treatment groups (see **Table 1** for details). For details of Protocols 1 and 2, see schematic in **Figure 1**). An additional two litters of pups were administered valproate for serum analysis of drug levels. Researchers were blinded to the treatment groups for all analyses reported here.

**Table 1:** Numbers of ligated mice for each set of experiments.

	Valproate	Saline	TSA	DMSO
**Groups for Protocol 1**
Assigned to each group, *n*	28	21	19	16
Mortality during treatment, *n*	5	2	3	2
Mortality after treatment, *n*	3	0	2	1
Surviving to perfusion, *n*	20	19	14	13
Stroke-injured at P60, *n* (%)	15 (75.0), 1 microinjured	15 (78.9), 4 microinjured	9 (64.3), 1 microinjured	10 (76.9)
Injured, *n*	15 (7 male)	15 (6 male)	9 (4 male)	10 (5 male)
Uninjured, *n*	5 (4 male)	4 (4 male)	5 (3 male)	3 (1 male)
**Groups for Protocol 2**
Assigned to each group, *n*	21	20	16	10
Mortality during treatment, *n*	4	2	1	1
Mortality after treatment, *n*	2	0	0	0
Surviving to perfusion, *n*	15	18	15	9
Stroke-injured at P42, *n* (%)	8 (53.3)	9 (50.0)	10 (66.7)	4 (44.4)
Injured, *n*	8 (5 male)	9 (3 male)	10 (3 male)	4 (3 male)
Uninjured, *n*	7 (1 male)	9 (7 male)	5 (3 male)	5 (1 male)

### ISCHEMIC STROKE AT P12

Newly born litters of pups arrived at postnatal 5 days old and were allowed to acclimate for 7 days. On P12, animals were subjected to permanent unilateral double ligation of the carotid artery, adapted from previous studies (Comi et al., 2004). In brief, animals were anesthetized with isoflurane carried by a 50–50 mixture of O_2_ and NO. The right common carotid artery was double ligated with 6-0 surgisilk and the outer skin closed with 6-0 monofilament nylon. Sham control animals were treated identically except the carotid was neither dissected nor ligated.

### ACUTE SEIZURE SCORING

Seizure activity was scored according to a seizure rating scale as previously reported. Immediately following ligation or sham surgery, pups were kept in an incubator at 36.5–37°C for 4 h during which seizure activity was monitored (Kadam et al., 2010). After 4 h, the mice were returned to the dam and each of their seizure scores was individually summed to produce a total acute seizure score. In these experiments, since seizure severity has been repeatedly shown to correlate with severity of brain injury in this model, seizure scores were used to help distribute injured animals as evenly as possible between the treatment groups. Mice with similar seizure score severities were randomly assigned to each treatment group.

### ANTICONVULSANT TREATMENT

Drug or vehicle was administered twice daily at 9 am and 5 pm by intraperitoneal injections for 2 weeks, and then weaned off over 2 days (see schematic in **Figure 1**). Valproic acid sodium salt (Cat # P4543, Sigma, St. Louis, MO, USA) was dissolved in sterile water for injections in working solution concentrations of 100 mg/ml and injected at 200 mg/kg. Saline was injected as the vehicle control for valproate. TSA (Cat# met-tsa-5, Invitrogen) was reconstituted in a 5% DMSO solution in a concentration of 0.5 mg/ml and injected at 2.5 mg/kg. 5% DMSO (in saline) was injected as the vehicle control for TSA.

### SERUM COLLECTION AND ANALYSIS

Serum was collected for analysis of valproate concentration. Two litters (*n* = 20 mice; 3 died before collection of blood) received intraperitoneal injections of valproate twice daily, along the same time-scale as other mice, although without ligations or any bromodeoxyuridine (BrdU) injections. On P26, animals were assigned to have blood collected during either a time-point before the morning injections (to represent trough levels), an hour after morning injections (to represent peak levels), before evening injections (trough levels), or an hour after evening injections (peak levels). Mice were exposed to CO_2_ for 2 min or until breathing ceased. The ribcage was then opened and the heart exposed. Blood was collected by syringe via a cardiac puncture. It was left to sit at room temperature for an hour, and then put in a centrifuge at 4°C to spin for 15 min at 14000 *g*. The serum was then aspirated off and sent to the Auburn University Clinical Pharmacology laboratory for analysis of drug concentration.

### BROMODEOXYURIDINE LABELING AND TISSUE PREPARATION

Bromodeoxyuridine (BrdU; Sigma) reconstituted in 0.9% NaCl was intraperitoneally injected (five injections; 50 mg/kg) at P20–22 in Protocol 1 and P24–26 in Protocol 2. All the mice in the study were anesthetized with chloral hydrate (90 mg/ml; intraperitoneally) before being transcardially perfused with PBS followed by phosphate buffered 10% formalin, then having brains removed and post-fixed in the same fixative for 5 days. The brains were cryoprotected in sucrose after which they were rapidly frozen using dry ice and placed in -80°C storage. Coronal brain sections 40 μm thick were cut on a cryostat in serial order to create six series of sections that were mounted on super frost plus glass slides and stored at -20°C.

### COMPUTERIZED BRAIN ASYMMETRY ANALYSIS

Using MCID 7.0 Elite (InterFocus Imaging Ltd, Cambridge, UK) brain atrophy scores (of the affected right side compared to the contralateral side) were measured for evenly spaced Nissl stained brain sections spanning between the levels of the anterior horn of the lateral ventricle and the caudal hippocampus as previously described. For each brain, hippocampal, and hemispherical atrophy scores from a series of equidistant sections were combined to calculate average atrophy scores as previously described (Markowitz et al., 2011).

### IMMUNOHISTOCHEMISTRY

The following primary antibodies were used: mouse anti-BrdU (1:200, BMC9318, Roche Applied Sciences, Indianapolis, IN, USA), mouse anti-NeuN (1:2000, MAB377 Chemicon, Temecula, CA, USA), rabbit anti-NeuN (1:1000, ABN78, Chemicon). Antigen retrieval was performed with citrate buffer (pH 6.0), and then DNA denaturated with HCL (2 mol/l), after which slides were neutralized with 0.1 M borate buffer (pH 8.5). After blocking non-specific binding sites, primary antibodies were applied. Anti-BrdU and anti-NeuN were then linked to Alexa Fluors 488 and 594 (1:400, Invitrogen), respectively.

### NEUROGENESIS ASSESSMENT

**FIGURE 2:**
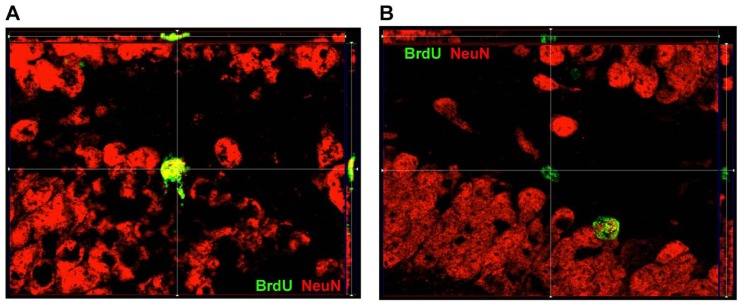
**ApoTome image of localization of BrdU and NeuN within DG. (A)** Representative image of a cell within SGZ that is BrdU+ and expressed NeuN as well. **(B)** Representative image of a cell within SGZ that is BrdU+ but does not express NeuN.

Counts were performed with an Olympus BX61 microscope equipped with filter cubes for visualizing fluorophores absorbing in blue, green, and red ranges. SGZ was defined as the two-cell thick layer between the GCL and hilus. Cells on the border between SGZ and GCL were included in SGZ counts if greater or equal to half of the cell was within the SGZ area. Cell counts were done in five consecutive sections per brain series (coordinates of which ranged roughly from Bregma -1.46 to -2.70 mm; Paxinos and Franklin, 2001) and average cell densities calculated for each area bilaterally. Thus 100% sampling (i.e., shown to be equivalent to a sampling grid of counting frames adjacent to one another covering the entire regions of interest, ROI; Crews et al., 2004) was done through entire ROIs in the maximum number serial coronal sections (*n* = 5) in which the ROIs could be clearly defined in the ipsilateral injured hemisphere (Manaye et al., 2007) by closed contours. This method, a modification of systematic random sampling was done as described previously by Manaye et al. (2007), where every cell of interest (i.e., BrdU positive cell) in the ROIs was counted. We started from section at which both DG blades are formed and included all cells that expressed BrdU. Our method allowed for cycling through the *z*-axis, live visualization under a filter that showed green and red channels simultaneously (for co-labeling which was confirmed by images obtained with an Zeiss Imager. M2 with an ApoTome attachment, see **Figure 2**), as well as live visualization in single channels in order to ascertain whether labeling was within the same cell or labeling of overlapping cells. Density of newly born cells in the GCL was determined by dividing the number of BrdU-labeled cells co-labeled with NeuN, by the area of the GCL as measured by MCID. Density of newly born neural cells in the SGZ was determined by dividing the number of BrdU-labeled cells co-labeled with NeuN, by the length of the SGZ as measured by MCID. Counts of cells in the GCL were unique and non-inclusive of the cells seen in the SGZ. Additionally, counts were obtained for the hilus, defined as the area between the two blades of the granule cell layer, excluding cells in the SGZ. Percentages for co-labeling for GCL and SGZ were determined by the following formula: [(BrdU+NeuN+ cell density)/(BrdU+ cell density)] × 100.

### BEHAVIORAL TESTING

#### Open field test

***Apparatus.*** Open field assessments were carried out in square fields (40.6 cm × 40.6 cm, Accuscan, Columbus, OH, USA) that are mounted within specially designed sound attenuating chambers constructed of polypropylene and polyvinyl chloride (PVC). The fields were illuminated only by dim red light (6 V). Behavior was monitored via a grid of invisible infrared light beams mounted on the sides of the walls of the arena and equally spaced from the front to back and from left to right. Data was collected and analyzed via VersaMax Analyzer software (Accuscan, Columbus, OH, USA), which is capable of determining the position of a mouse 50 times per second.

***Procedure.*** To examine activity levels and habituation, mice were exposed to the test chambers for 30 min on each of two consecutive days.**To begin a session, each mouse was placed in the center of the chamber and allowed to move about freely. The arena was cleaned with 70% ethanol after each mouse completed a session.

### T-MAZE SPONTANEOUS ALTERNATION

#### Apparatus

This procedure was carried out in an enclosed “T” shaped maze (Med Associated, St. Albans, VT, USA) in which the long arm of the T (47 cm × 10 cm) serves as a start arm and the short arms of the T (35 cm × 10 cm) serve as the goal arms.

#### Procedure

In this task the mouse was placed in the start arm and after 5 s the door was opened and the mouse was allowed to choose and explore one of the goal arms. When the mouse had fully entered the choice arm (tail tip all the way in) the arm was closed and the mouse was confined to the choice arm for 30 s. The mouse was then removed, the guillotine door lifted and the next trial initiated. This was repeated for a total of 15 trials. If the mouse did not make a choice within 2 min the trial was ended and advanced to the next. At the conclusion of each trial the maze was cleaned with 70% ethanol to eliminate odors.

### NOVEL OBJECT PREFERENCE

#### Apparatus

Novel object preference assessment is carried out in the same chambers as the open field above.

#### Procedure

The novel object preference was carried out over 2 days immediately following the open field test described above. On day 1 of the preference test mice were placed in the open field described above and allowed to move about freely for 30 min. As this session follows the prior two sessions in the open field, the additional session during the preference test serves to further habituate the mice to the chambers and ensure that the chamber environment is no longer novel. On day 2 of the preference test, mice were again placed in the chambers and exposed to various objects (plastic strawberry, miniature flower pot, metal star, or small stone) to assess novel object preferences. This took place in two stages, a sample phase and a test phase. During the sample phase, two identical objects were mounted to the floor of the open field on opposite sides of the arena and equidistant from the walls. Mice were placed into the center of the open-field, and allowed 10-min to explore. At the end of the sample phase, each mouse was returned to the home cage for a 3-min interphase interval (IPI) during which one of the objects was replaced with a different object. Following the IPI, mice were returned to the field and given a 10-min test phase. The time each mouse spent exploring each object during each phase was recorded and this measure serves as an index of object preference.

### METHODS OF ANALYSIS

Statistical analyses were run in SPSS for Windows (SPSS Inc., Chicago, IL, USA) and SYSTAT 12 (SYSTAT software, Inc., Chicago, IL, USA). ANOVAs were carried out to analyze behavioral data and weight data. Independent sample T tests were carried out to analyze the area and count data for the ipsilateral and contralateral sides in the ligation-injured group comparing treatment groups. Poisson regression was performed in rostral to caudal analysis of neurogenesis data by section to determine the overall significance of treatment effects. Gender differences were noted where relevant. A probability below 0.05 was considered significant. All mean values are presented ± standard error of the means. Fisher’s exact tests were done to assess mortality and data from mice that died were excluded from all other analyses.

To examine overall locomotor activity and habituation, mice were observed in an open-field over 2 consecutive daily sessions. Analysis of all between session open field data was performed on only the first half of each session (first 15 min) because all of the mice were the most active during this period and activity declined substantially by the second half. Total distance traveled and rearing activity served as indices of overall activity. Open field data was analyzed by ANOVAs in which the between session open field data were distance traveled and rearing, where Condition (Valproate, Saline, and Sham, or TSA, DMSO, and Sham) served as the between subjects factor, and were day (day 1 session versus day 2 session) served as the within subjects factor. Analysis of within session open field measures included Condition as a between subjects factor and Time Block (5 min blocks of time) as the within subjects factor. Analysis of rotational movements in the open field included Condition as a between subjects factor and rotation (clockwise and counter-clockwise movements for day 1 session only) as the within subjects factors. Spontaneous alternation on the T-maze and the novel object preference data were analyzed with independent sample *t*-tests. There were no performance differences between male and females on any of the behavioral tests, thus analysis was carried out collapsed across gender. A total of five ligation-injured mice (two TSA, one DMSO, one Valproate, and one saline) were excluded from analysis of the open field data as extreme outliers due to a fourfold increase in locomotion, and a predominant clockwise wild running behavior demonstrated by the mice on one or both days of testing. This abnormal behavior has been previously described in this model (Kadam et al., 2010), and is associated with severe brain injury and the development of chronic behavioral seizures.

Of the mice that were ligated, only those that showed injury were included in the analyses reported here (see **Table 1**). In all cases where the injury was not obvious, microscopic examination of cresyl violet-stained sections was done looking for focal atrophy, gliosis, and cell loss. The few micro-injured brains (see **Table 1**) were excluded from neurogenesis and behavioral analyses because our prior studies have shown that this very mild form of injury impact neurogenesis differently than the more severe stroke injury.

## RESULTS

### VALPROATE TREATMENT

#### Seizure scores

In Protocol 1, seizure scores in injured animals ranged from 0 to 41 (median = 0) in the valproate-treated and 0 to 66 (median = 0) in the saline-treated animals (N.S.). In Protocol 2, seizure scores ranged from 0 to 47 (median = 12) in the valproate-treated and 0 to 120 (median = 2) in the saline-treated (N.S.). Seizures correlated with hemispheric brain atrophy in both valproate (*r*^2^ = 0.566, *p* = 0.006) and saline treated animals (*r*^2^ = 0.463, *p* = 0.040).

#### Treatment groups and associated mortality

The sample size of valproate or saline treated ligated mice in Protocol 1 was *n* = 28 valproate- and 21 saline-treated (see **Table 1** for details). Compared to saline (2/21 died), mortality associated with the valproate treatment (8/28 died) is not significantly higher (Fisher’s exact *p* = 0.16). The sample size for Protocol 2 is 21 ligated valproate- and 20 saline-treated ligated mice. Compared to saline (2/20 died), mortality associated with the valproate treatment (6/21 died) is higher but not significantly so (Fisher’s exact, *p* = 0.24). When the mortality data from the first 42 days of both protocols are added together, the mortality in the valproate-treated group is significantly higher compared to saline-treated ligated animals (Fisher’s exact, *p* = 0.035).

#### Impact upon weight gain

**FIGURE 3:**
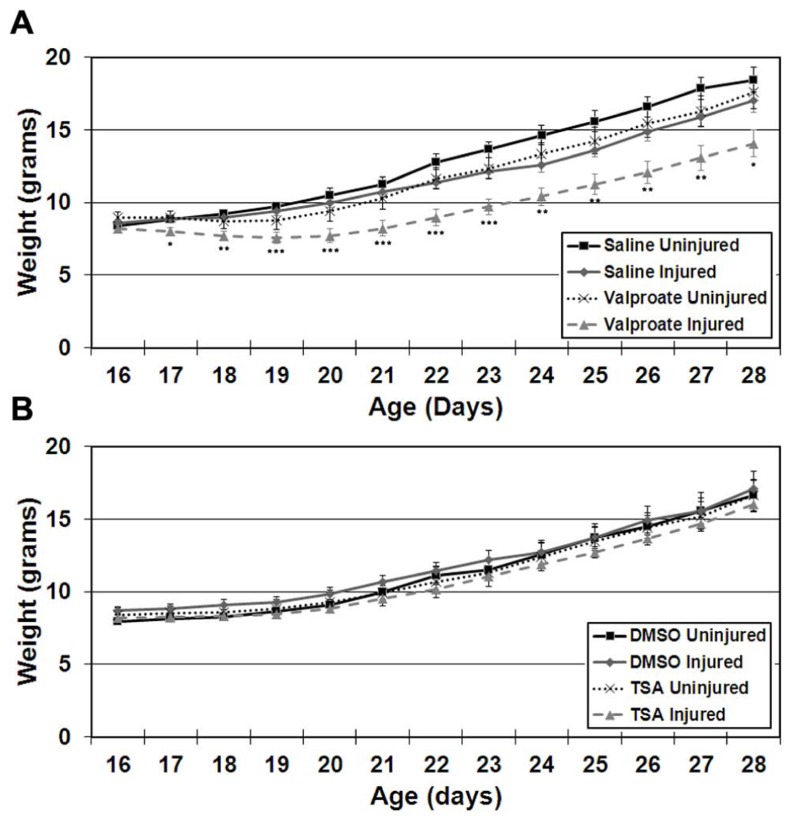
Weight gain starting on the day of commencement of treatment (P16) until cessation of treatment (P28) in ligation-uninjured and ligation-injured mice (A) treated with valproate or saline-vehicle (Protocol 2) or (B)TSA or DMSO-vehicle (Protocol 2; *p < 0.05, **p < 0.01, ***p < 0.001).

Stroke-injured mice treated with valproate had lower weights compared to saline-treated injured mice in both females and males (**Figure 3A**; repeated measures ANOVA, *p* = 0.037). This difference in weight occurred in both Protocols 1 and 2 and resolved after discontinuing the valproate treatment (weight at P60 in valproate-treated = 27.9 ± 1.7 g and in saline-treated = 29.0 ± 1.7 g, N.S., Protocol 1 and weight at P42 in valproate-treated = 24.8 ± 1.8 g and in saline-treated = 23.8 ± 1.3 g, N.S., Protocol 2).

#### Impact on brain atrophy

**FIGURE 4:**
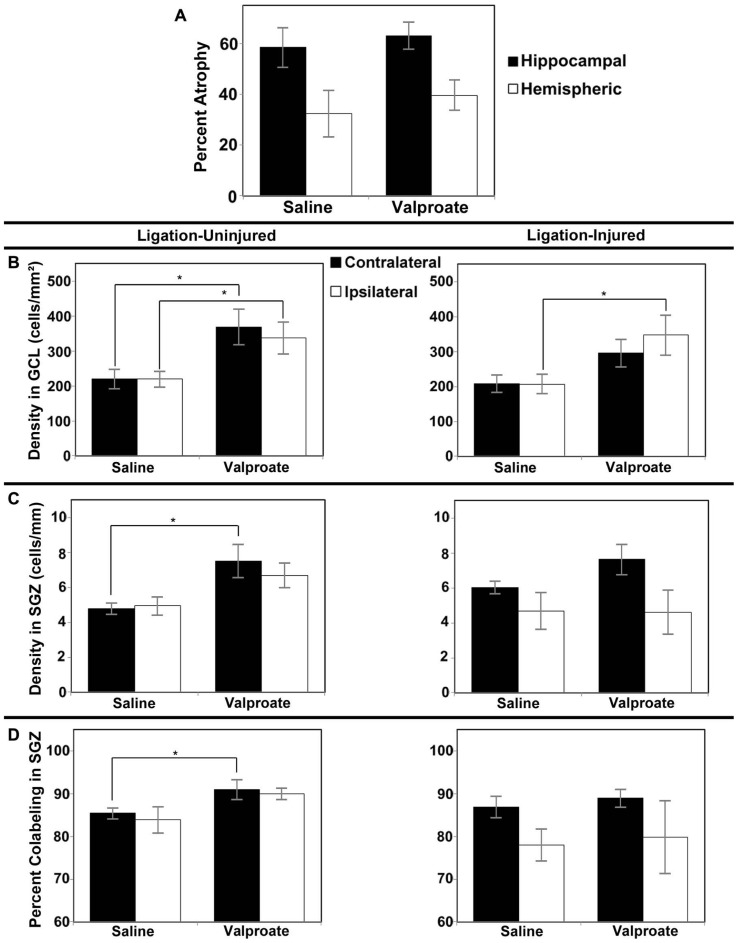
**Brain atrophy and dentate gyrus neurogenesis after valproate treatment in Protocol 2.** **(A)** Severity of hemispheric and hippocampal brain atrophy following P12 ligations quantified at P42 in ligation-injured mice. **(B) **Average densities of BrdU+/NeuN+ within the GCL in both ligation-uninjured (left panel) and ligation-injured (right panel) mice treated with valproate or saline. **(C) **Average densities of BrdU+/NeuN+ cells along the SGZ in both ligation-uninjured (left panel) and ligation-injured (right panel) mice treated with valproate or saline. **(D)** Percent of BrdU-positive cells along the SGZ that express NeuN in both ligation-uninjured (left panel) and ligation- injured (right panel) mice treated with valproate or saline (*p < 0.05).

In Protocol 1 compared to its vehicle control group, in the valproate-treated group of ligation-injured mice there was no significant change in stroke-injury atrophy at P60 (valproate: 32.5 ± 5.0% hemispheric and 53.2 ± 5.9% hippocampal atrophy versus saline: 25.5 ± 4.8% hemispheric and 50.3 ± 6.3% hippocampal; *p* = 0.335 and 0.734, respectively). Similarly at P42 (Protocol 2), there were no significant differences in brain atrophy (see **Figure 4A**). No sex related differences within each group were found to be significant for severity of brain atrophy at P42 or at P60.

#### Impact on neurogenesis

Comparing the neurogenesis in the GCL, SGZ and hilus, as measured above, in both valproate- and vehicle-treated sham surgery mice, to that in the contralateral DG of uninjured ligated mice, there were no significant differences (Protocol 1, data not shown). Since neither valproate nor vehicle treated sham surgery animals were significantly different in their neurogenesis from the uninjured ligated animals, uninjured ligated animals were used as controls for the neurogenesis studies; this prevented the use of additional animals in Protocol 2. Protocol 1 treatment with valproate starting at P16 (i.e., 4 days after stroke-insult) and BrdU labeling of proliferating cells at P20–22 (i.e., after 4–6 days of valproate exposure) did not show any significant effect on DG neurogenesis in the dorsal hippocampus in stroke-uninjured mice (mean density of BrdU labeled cells in contralateral GCL + SGZ in valproate = 813 ± 208 cells/mm^2^ versus saline = 533 ± 66 cells/mm^2^, *p* = 0.259; mean ipsilateral density in valproate = 828 ± 205 cells/mm^2^ versus saline = 520 ± 73 cells/mm^2^, *p* = 0.241) or in the stroke-injured mice (mean contralateral density in valproate = 621 ± 58 cells/mm^2^ versus saline = 605 ± 40, *p* = 0.829 cells/mm^2^; mean ipsilateral density in valproate = 620 ± 129 cells/mm^2^ versus saline = 448 ± 52 cells/mm^2^, *p* = 0.303).

However, with administration of the BrdU later, from P24–P26, and assessing BrdU–NeuN co-labeling at P42, increased density of co-labeled cells was noted when comparing valproate-treated mice to saline-treated counterparts. Valproate-treated injured GCL new cell density was significantly increased relative to controls ipsilaterally (*p* = 0.046) and a trend was noted for increase contralaterally (*p* = 0.074; see **Figure 4B**). Valproate similarly significantly increased new cell densities in ligation-uninjured mice in ipsilateral and contralateral GCL (*p* = 0.027 and 0.016, respectively; see **Figure 4B**). There were no significant differences in percent of cells labeled with BrdU expressing NeuN within GCL, when comparing valproate-treated or saline-treated injured or uninjured mice as they were all upward of 99%.

In the SGZ, there was a trend noted for increased new cell density in valproate-treated ligation-uninjured mice ipsilaterally (*p* = 0.063), while there was a significant increase contralaterally (*p *= 0.028; see **Figure 4C**). In ligation-injured animals, there was no significant difference in new cell density in SGZ between valproate and saline-treatment ipsilaterally (*p* = 0.964) while there was a trend noted for increased cell density in valproate-treated animals contralaterally (*p* = 0.095; **Figure 4C**). In uninjured mice, there was also an increase in the percent of cells labeled with BrdU along the SGZ that also expressed NeuN among valproate-treated animals compared to saline-treated, contralaterally (*p* = 0.041) although the increase was not significant ispilaterally (*p* = 0.125; **Figure 4D**). In injured mice, there was no significant difference between the two treatments in the percent of cells co-labeled either contralaterally or ipsilaterally (*p* = 0.533 and 0.850, respectively; **Figure 4D**).

There were no significant differences noted in densities of new BrdU–NeuN co-labeled cells in the hilus after treatment with valproate or saline in either ligation-injured or uninjured animals. In injured mice, contralateral mean count valproate = 0.33 ± 0.06 cells versus saline = 0.50 ± 0.12 cells, *p* = 0.243; and ipsilateral mean count valproate = 0.28 ± 0.10 cells versus saline = 0.49 ± 0.13 cells, *p* = 0.232. In uninjured mice, contralateral mean count valproate = 0.31 ± 0.07 cells versus saline = 0.42 ± 0.15 cells, *p *= 0.533; and ipsilateral mean count valproate = 0.54 ± 0.11 cells versus saline = 0.56 ± 0.11 cells, *p* = 0.938. No significant sex differences were noted in neurogenic responses.

### TRICHOSTATIN A TREATMENT

#### Seizure scores

In Protocol 1, seizure scores ranged from 0 to 75 (median = 0) in the TSA-treated and 0 to 104 (median = 0) in the DMSO-treated animals (N.S.). In Protocol 2, seizure scores ranged from 0 to 21 (median = 0) in the TSA-treated and 0 to 0 (median = 0) in the DMSO-treated (N.S.). Seizures correlated with hemispheric brain atrophy in both TSA- (*r*^2^ = 0.560, *p* = 0.016) and DMSO-treated animals (*r*^2^ = 0.682, *p* = 0.007).

#### Treatment groups and associated mortality

The sample size of TSA- or DMSO-treated ligated mice in Protocol 1 was *n* = 19 TSA- and 16 DMSO-treated (see **Table 1** for details). Compared to DMSO (3/16 died), mortality associated with the TSA-treatment (5/19 died) was not significantly higher (Fisher’s exact *p* = 0.700). The sample size for ligated mice in Protocol 2 was 16 TSA-treated and 10 DMSO-treated mice. Compared to DMSO (1/10 died), mortality associated with the TSA-treatment (1/16 died) was not significantly higher (Fisher’s exact *p* = 1.000). When the mortality data from the first 42 days of both protocols are added together, the mortality in the TSA-treated group is still not significantly higher compared to DMSO-treated ligated animals (Fisher’s exact *p* = 1.000).

#### Impact upon weight gain

Stroke injured mice treated with TSA did not exhibit altered weight gain compared to DMSO-treated injured mice in either females or males (**Figure 3B**).

#### Impact on brain atrophy

**FIGURE 5:**
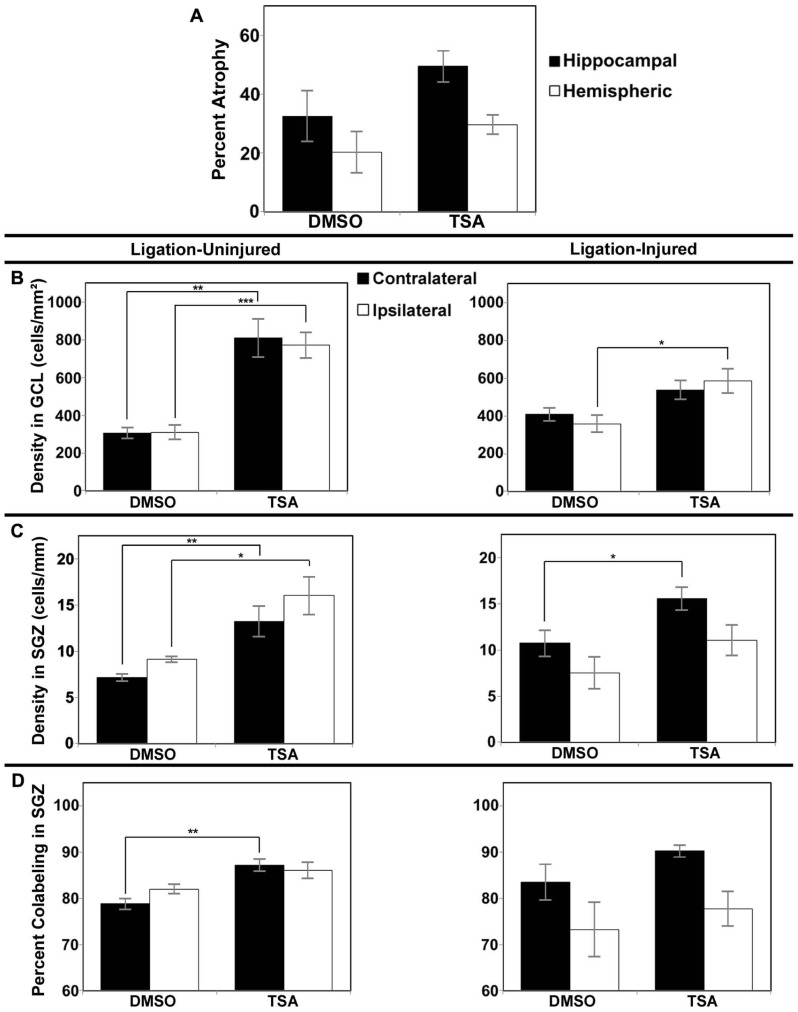
**Brain atrophy and dentate gyrus neurogenesis afterTSA treatment in Protocol 2.** **(A)** Severity of infarct injury following P12 ligations quantified at P42 (14 days following cessation of treatment with TSA or DMSO-vehicle at P28) in ligation-injured mice. **(B) **Average densities of BrdU+/NeuN+ within the granule cell layer (GCL) in both ligation-uninjured (left panel) and ligation-injured (right panel) mice treated with TSA or DMSO. **(C) **Average densities of BrdU+/NeuN+ cells along the subgranular zone (SGZ) in both ligation-uninjured (left panel) and ligation-injured (right panel) mice treated with TSA or DMSO. **(D)** Percent of BrdU-positive cells along the SGZ that express NeuN in both ligation-uninjured (left panel) and ligation-injured (right panel) mice treated with TSA or DMSO (*p < 0.05, **p < 0.01, ***p < 0.001).

In Protocol 1 the stroke-injury related atrophies in the DMSO-treated group was very similar to the TSA-treated group of ligation-injured mice (34.9 ± 7.2 and 37.3 ± 7.5% hemispheric atrophy, respectively, and 57.6 ± 8.3 and 59.6 ± 6.6% hippocampal atrophy, respectively; *p *= 0.821 and 0.858, respectively). Similarly at P42 (Protocol 2) there were no significant differences in brain atrophy (**Figure 5A**; DMSO = 20.2 ± 6.9% hemispheric and 32.5 ± 8.6% hippocampal atrophy; TSA = 29.6 ± 3.3% hemispheric and 49.5 ± 5.4% hippocampal atrophy; *p* = 0.190 and 0.118, respectively). No gender related differences within each group were found to be significant for severity of brain atrophy at P42 or at P60.

#### Impact on neurogenesis

Protocol 1 treatment with TSA also demonstrated no change in neurogenesis within treatment groups compared to DMSO-treated controls in stroke-uninjured (mean density of BrdU labeled cells in contralateral GCL + SGZ in TSA = 770 ± 109 cells/mm^2^ versus DMSO = 779 ± 69 cells/mm^2^, *p *= 0.956; mean ipsilateral density in TSA = 719 ± 104 cells/mm^2^ versus DMSO = 676 ± 57 cells/mm^2^, *p* = 0.777) or in the stroke-injured mice (mean contralateral density in TSA = 642 ± 52 cells/mm^2^ versus DMSO = 542 ± 60, *p* = 0.240; mean ipsilateral density in TSA = 495 ± 72 cells/mm^2^ versus DMSO = 498 ± 55 cells/mm^2^, *p* = 0.974).

However, compared to vehicle-treated injured controls, Protocol 2 treatment with TSA for 9 days before BrdU labeling, resulted in increases in DG neurogenesis (determined as cell density or as cell counts; density reported here) in animals treated with TSA (2.5 mg/kg). One TSA-treated injured animal had excessively high density of DG BrdU–NeuN co-labeled cells bilaterally (greater than four times the mean) and was excluded from this analysis. In ligation-injured mice, new cell density was significantly higher with TSA-treatment in ipsilateral GCL (*p* = 0.045), but the increase was not significant in contralateral GCL (*p* = 0.139; see **Figure 5B**). With TSA-treatment in uninjured animals, new cell density was significantly increased in GCL in both contralateral and ipsilateral hemispheres (*p* = 0.002 and 0.0003, respectively; **Figure 5B**). No differences were noted between percent of cells labeled with BrdU within GCL that express NeuN when comparing TSA-treated with DMSO-treated injured or uninjured mice as they were all above 99%.

In ligation-injured mice, mean new cell density along the SGZ was significantly increased contralaterally in mice treated with TSA (*p* = 0.045) although the increase was not significant ipsilaterally (*p* = 0.232; **Figure 5C**). In ligation-uninjured mice, new cell density was significantly elevated along the SGZ in both hemispheres with TSA-treatment (contralateral, *p* = 0.007; ipsilateral, *p* = 0.010; **Figure 5C**). Similarly to the effects of valproate, after treatment with TSA, there were higher percentages of BrdU+ cells in SGZ that co-labeled with NeuN. In ligation-uninjured mice, there was a significant increase in contralateral SGZ (*p* = 0.002), but only a trend for increase in ipsilateral SGZ (*p* = 0.081; **Figure 5D**). In ligation-injured mice, there was a trend for increased percent co-labeling in contralateral SGZ (*p* = 0.054), but no significant increase in ipsilateral SGZ (*p* = 0.523).

No significant differences were noted in counts of BrdU–NeuN co-labeled cells in the hilus corresponding with TSA or DMSO treatment in ligation-injured animals (contralateral mean count TSA = 1.04 ± 0.21 cells versus DMSO = 1.30 ± 0.34 cells, *p* = 0.524; and ipsilateral mean count TSA = 0.96 ± 0.25 cells versus DMSO = 1.05 ± 0.29 cells, *p* = 0.827). In ligation-uninjured animals, contralateral mean counts in mice treated with TSA = 1.56 ± 0.16 versus DMSO = 0.92 ± 0.17, *p* = 0.027; and ipsilateral mean counts in TSA = 1.96 ± 0.59 versus DMSO = 0.96 ± 0.23, *p* = 0.172.

#### Poisson regression of neurogenesis

**FIGURE 6:**
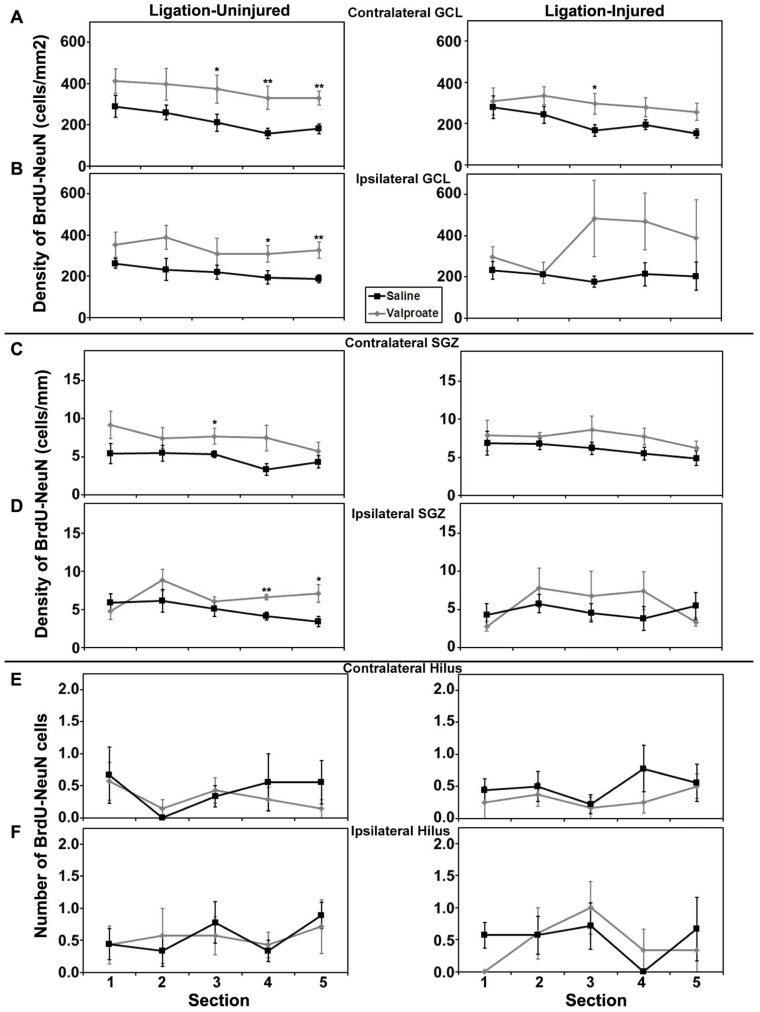
**Aerial analysis (rostral to caudal) of BrdU–NeuN density (A–D) or counts (E,F) in mice treated with valproate or saline in Protocol 2. (A,B)** Average densities of BrdU+/NeuN+ within the contralateral **(A)** and ipsilateral **(B)** GCL in both ligation- uninjured (left panel) and ligation-injured (right panel) mice treated with valproate or saline. **(C,D) **Average densities of BrdU+/NeuN+ cells along the contralateral **(C)** and ipsilateral **(D)** SGZ in both ligation- uninjured (left panel) and ligation-injured (right panel) mice treated with valproate or saline. **(E,F)** Average counts of BrdU+/NeuN+ cells in the contralateral **(E)** and ipsilateral **(F)** hilus in both ligation-uninjured (left panel) and ligation-injured (right panel) mice treated with valproate or saline (*p < 0.05, **p < 0.01).

**FIGURE 7:**
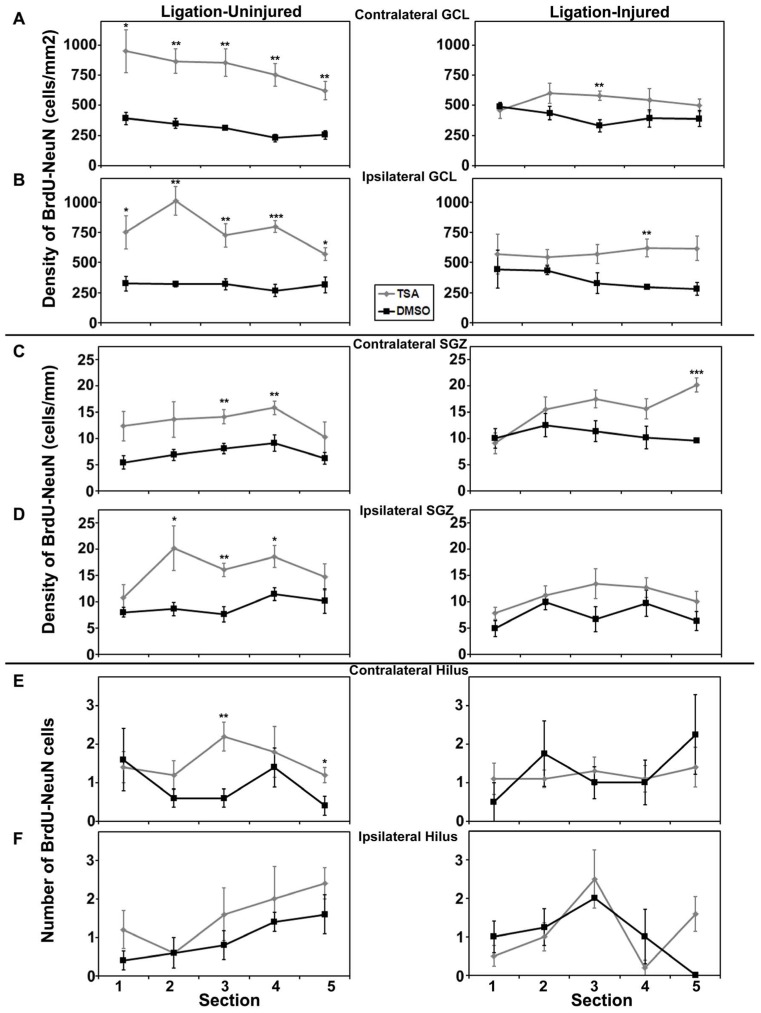
**Aerial analysis (rostral to caudal) of BrdU–NeuN density (A–D) or counts (E,F) in mice treated withTSA or DMSO in Protocol 2. (A,B)** Average densities of BrdU+/NeuN+ within the contralateral **(A)** and ipsilateral **(B)** GCL in both ligation-uninjured (left panel) and ligation-injured (right panel) mice treated with TSA or DMSO. **(C,D) **Average densities of BrdU+/NeuN+ cells along the contralateral **(C)** and ipsilateral **(D)** SGZ in both ligation-uninjured (left panel) and ligation-injured (right panel) mice treated with TSA or DMSO. **(E,F)** Average counts of BrdU+/NeuN+ cells in the contralateral **(E)** and ipsilateral **(F)** hilus in both ligation-uninjured (left panel) and ligation-injured (right panel) mice treated with TSA or DMSO (*p < 0.05, **p < 0.01, ***p < 0.001).

Rostral to caudal analysis by section did not elucidate any differences in patterns of distribution of neurogenesis in either GCL or SGZ in animals treated with valproate, TSA or their vehicles. In uninjured animals treated with TSA versus DMSO, analysis by section revealed a pattern of increased counts rostral to caudal in contralateral hilus (*p* = 0.034) and a trend for the same in ipsilateral hilus (*p* = 0.052). This pattern of counts in the hilus by section was not seen in injured animals treated with TSA or DMSO, or any animals treated with valproate or saline (see **Figures 6** and **7**).

#### Cognitive testing

**FIGURE 8:**
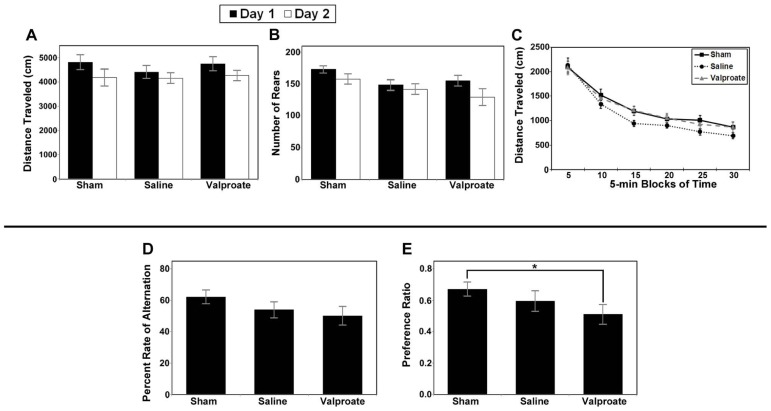
**Results of behavioral studies in valproate- and saline-treated injured mice compared to sham controls in Protocol 1. (A–C)** Open field studies demonstrated no significant differences in distance traveled, rearing behavior or habituation.** (D) **T-maze alternation test demonstrated no significant differences between the groups.** (E) **Ligation-injured mice receiving valproate performed less well on novel object testing compared to sham controls (*p < 0.05).

Analysis of total distance traveled for the valproate group (i.e., injured mice given valproate, their injured saline-controls and the shams) revealed only a main effect of day (*F*_1,34_ = 16.743, *p* < 0.001; **Figure 8A**) due to an overall decrease in ambulation from day 1 to day 2 (habituation). Analysis of the rearing data for this group of animals also revealed a main effect of day (*F*_1,34_ = 8.258, *p* = 0.007; **Figure 8B**). To evaluate within session habituation, the total distance traveled during the entire 30-min session was analyzed in 5-min blocks for each day. Analysis of the valproate group revealed only a significant main effect of block for both sessions (*F*_5,170_ = 169.753, and *F*_5,170_ = 172.389, *p* < 0.001; **Figure 8C**). Analysis of the data for the injured valproate -treated group of mice did not reveal any significant effects for the percent alternation rate (see **Figure 8D**), percent right turns made, or number of trials completed. The overall analysis of the novel object preference task data for the valproate group of mice did not reveal any significant differences. However, given the apparent low preference score of the valproate ligation-injured mice (≈0.5, indicating no preference), we constructed a simple contrast to compare the preference ratio between the valproate and sham mice, and a significant *P*-value was obtained (*t*_25_ = 2.132, *p* = 0.043; **Figure 8E**) suggesting impaired recognition memory in the valproate-treated ligation-injured mice. The failure to obtain a significant effect with the omnibus ANOVA likely reflects insufficient power to detect a difference.

**FIGURE 9:**
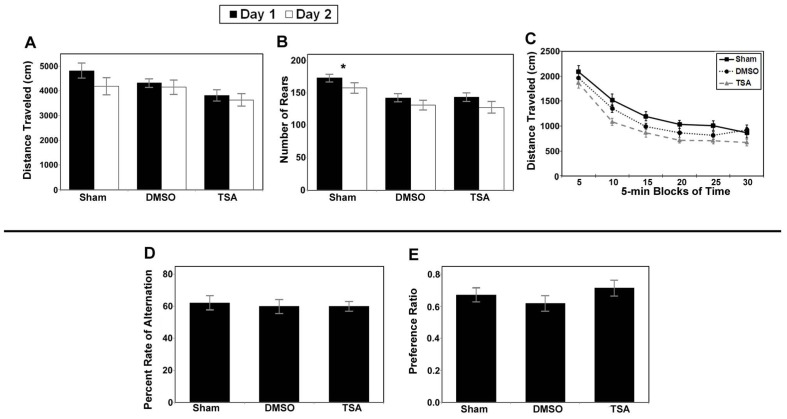
**Results of behavioral studies inTSA- and DMSO-treated injured mice compared to sham controls in Protocol 1.** **(A–C) **Open field studies demonstrated no significant differences in distance traveled and habituation, however the injured animals in both treatment groups reared less than the shams.** (D) **T-maze alternation test demonstrated no significant differences between the groups.** (E) **Novel object testing also demonstrated no significant differences (*p < 0.001).

Similar to the valproate group, analysis of the total distance traveled by the TSA group (i.e., injured mice given TSA, their injured DMSO-controls, and shams) also yielded a significant main effect of day (*F*_1,34_ = 7.148, *p* = 0.011; **Figure 9A**). However, in contrast to the valproate group, analysis of the rearing data for the TSA group yielded a significant main effect of Condition (*F*_2,34_ = 7.190, *p* = 0.002) in addition to the main effect of day (*F*_1,34_ = 12.012, *p* = 0.001; **Figure 9B**). The effect of Condition is due to the shams rearing more than either the TSA- or DMSO-treated mice; there were no differences between the TSA- and DMSO-treated on this measure. The main effect of day was again due to habituation across the sessions. Analysis of within session habituation in the TSA group of mice also yielded only significant effects for Block for both the day 1 session (*F*_5,170_ = 160.462, *p* < 0.001) and the day 2 session (*F*_5,170_ = 157.558, *p* < 0.001). These results illustrate that all mice habituated within each session on both days (see **Figure 9C**). There were no other main effects or interactions for the TSA group of mice.

Analysis of the data for the injured TSA group of mice did not yield any significant effects for the alternation rate (see **Figure 9D**), percent right turns made, or number of trials completed. There were also no effects for the number of trials completed by each group (TSA or controls). In addition, analysis did not reveal a significant difference in the preference ratio for novel objects (see **Figure 9E**).

Unilateral strokes are known to elicit circling movements in rodents (Kadam et al., 2010); this comorbidity worsens in frequency and severity over time and is associated with the development of hyperactivity seen in a subset of the animals. We therefore measured the clockwise and counter-clockwise rotations of all (non-hyperactive) mice during the open field sessions. However, the only significant differences we observed were main effects in clockwise rotations from day 1 to day 2 (TSA: *F*_1,34_ = 5.084, *p* = 0.031; valproate: *F*_1,35_ = 6.875, *p* =0.013) and counterclockwise rotations from day 1 to day 2 (TSA: *F*_1,34_ = 7.799, *p* = 0.009; valproate: *F*_1,35_ = 6.443, *p* = 0.016). These differences reflect the decrease in activity from day 1 to day 2 seen across all subjects and noted in the open field section above. There were no other differences either across or within each of the 2 days (in either the valproate- or the TSA-treated animals). This difference, compared to our prior study, may be due to the animals being studied at earlier time-points after the stroke injury, and the removal of hyperactive animals from the analysis.

#### Valproate serum analysis

Twenty mouse pups were administered valproate starting at P16, and three died before blood collection at P26. The mean afternoon peak level (*n* = 5) of valproate in serum was 241.9 ± 18.1 μg/ml. There were four samples for a corresponding trough level, of which three fell below levels of detection and one was 2.2 μg/ml. The mean peak level of valproate in serum after the morning dose (*n* = 4) was 264.0 ± 20.6 μg/ml while the mean trough level before the next dose (*n *= 4, one of which was below levels of detection) was 2.0 ± 0.3 μg/ml, resulting in a half-life of less than 1.0 h.

## DISCUSSION

In the present study, we determined whether administration of valproate, an anticonvulsant with multiple mechanisms of action including HDAC inhibition, and its positive control TSA, a potent broad HDAC inhibitor with no known anti-convulsant action, when given for almost 2 weeks during early life following a stroke, influences neurogenesis in the dorsal hippocampus of the maturing brain. After neonatal stroke, mice were administered the drugs twice daily from P16 to P28, but not during the immediate post-injury period, in order to avoid producing a secondary effect upon neurogenesis resulting primarily from an acute neuroprotective effect of the drug. The data presented demonstrate that (1) chronic HDAC treatment during this regenerative period did not modify the severity of stroke-related brain atrophy assessed at P60 or at P42, (2) BrdU labeling at 8–10 days post-stroke (4–6 days after either valproate or TSA initiation) showed no significant changes in DG neurogenesis compared to vehicle controls, (3) BrdU labeling at 12–14 days after stroke (7–9 days after either valproate or TSA initiation) revealed a significant increase in DG neurogenesis produced by TSA or valproate both in the injured and uninjured animals compared to the vehicle controls in both sexes and (4) valproate, but not TSA, was associated with an increase in mortality, impaired animal weight gain, and mild cognitive impairment.

Initially, we labeled with BrdU at P20–22 and waited approximately 4 weeks for the new granule cells to mature and be incorporated into circuits within the GCL. However, no differences were noted between drug and saline treatments. We had several hypotheses for why this may have been the case and designed Protocol 2 with these hypotheses in mind. The immature brain has a high level of neurogenesis compared with the more mature brain, which may reflect the highly plastic nature of the immature brain and its ability to learn (Kuhn et al., 1996). However it may also mean that this high level of plasticity and neurogenesis cannot be up-regulated any further by epigenetic manipulation; this situation may in part explain the lack of statistically significant increased neurogenesis noted in Protocol 1. As the animal ages and the neurogenesis falls below neonatal levels, however, induction of neurogenesis may become amenable by drugs that up-regulate the gene groups controlling neural development. In addition, the animal handling during behavioral testing in Protocol 1 likely also increased the post-stroke neurogenesis in all groups, thus making it more difficult to demonstrate a statistically significant increase in neurogenesis due to the HDAC inhibitors in Protocol 1.

On the other hand, it may also be possible that the 4–6 days of HDAC inhibition prior to administration of BrdU is insufficient to induce epigenetic changes that underlie SGZ neurogenesis in an immature brain; however, previous studies have shown up-regulation of neurogenesis with 3 day exposure of TSA in adult rats in a stroke model (Kim et al., 2009). Another possibility is that the evolving secondary inflammatory responses to the stroke injury (Faustino et al., 2011) may temporarily suppress HDAC activity producing a lack of response to HDAC inhibitors. The difference in neurogenic response between uninjured TSA-treated and injured TSA-treated mice supports this point, however a lack of a response in the uninjured animals in protocol 1, along with robust responses from uninjured animals in Protocol 2 argues against this being the sole factor at play. Finally, it is also possible that the increased neurogenesis noted with the BrdU administered in Protocol 2 (with survival to P42), but not when administered in Protocol 1 (with survival to P60), suggests that the HDAC inhibitors in these immature animals primarily increased SGZ proliferation and/or short-term survival and differentiation, rather than long-term survival, maturation, migration, and integration of the newborn SGZ granule cells. The small but significant increase in percent BrdU–NeuN co-labeling noted in the SGZ with HDAC inhibitor treatment supports this hypothesis. This result could represent an important alteration in the normal migration of these maturing neurons, or a reduction in normal loss of new-born neurons which do not properly migrate into the GCL. Future studies are needed determine which of these factors contribute to the different neurogenic responses seen when BrdU was administered under the two different protocols. Also, additional study is needed to determine the fate of the non-colabeled cells within the SGZ. Increased normal SGZ neurogenesis would be expected to reduce the risk of post-stroke epilepsy and cognitive impairments; however additional *abnormal* post-stroke and post-ischemic seizure neurogenesis could further contribute to abnormal hippocampal circuit formation and increase the risk of post-stroke morbidity (Jessberger et al., 2007). Importantly, treatment with TSA or valproate did not result in a change in newborn BrdU–NeuN co-labeled hilar or GCL neurons; alterations which would suggest aberrant migration of newborn neurons.

There was greater variability noted in densities of BrdU–NeuN cells in valproate-treated mice as compared to those in TSA-treated mice. The reason for this higher variability in valproate is not clear but may relate to non-CNS systemic effects of valproate on the liver or other mechanisms related to its toxicity and the effect of this on neurogenesis. It may also be noted that the neurogenesis seen with the saline and DMSO controls was different. DMSO may have some effect itself on neurogenesis, which is different from that noted with saline. There has been reported neurotoxicity associated with DMSO (Hanslick et al., 2009) that theoretically could affect neurogenesis differently as compared to saline. As the two drugs, valproate and TSA were dissolved in different solutions (saline and DMSO, respectively), it was important to have the proper controls for each of the drugs. The effects that these two vehicles have on neurogenesis could be different and therefore, it is vital to separate the effect of drug from that of vehicle. In the uninjured animals the effect of drugs upon the GCL was greater than their effect upon the SGZ; likely this is because at this timepoint many of the BrdU labeled cells have moved out of the SGZ and begun to differentiate. Because the effect of the drugs upon the SGZ was smaller, and given the variability in the results, it is likely that significance was not reached bilaterally in the SGZ measurement due to the number of animals used.

Trichostatin A did not produce any detected behavioral detriment in open-field, novel object preference or spontaneous alternation testing. Testing was done at P38–42; by this time newborn granule cells labeled at P20–22, or at P24–P26, have differentiated and migrated but are not expected to be fully matured and integrated (Kee et al., 2007). Recently it has been reported that 4–6 weeks is the time period required for adult neurogenesis to impact the novel object task (Denny et al., 2012). Full assessment of effects of increased neurogenesis, observed after BrdU labeling P24–26 and due to HDAC inhibition, may require additional testing of behavioral outcomes at about 2 months of age.

Valproate on the other hand may have contributed to mild cognitive impairments on novel object testing, although whether by HDAC inhibition or by another mechanism will require further study. Visual-spatial impairments in adult rats have been previously reported with prior chronic valproate-treatment (300 mg/kg twice a day for 10 days) and were associated with decreased SGZ proliferation (differentiation into neurons was not assessed) and decreased hippocampal BDNF and Notch 1 expression (Umka et al., 2010). In another study, however, no impact on SGZ proliferation, differentiation or survival was noted when 250 mg/kg valproate was administered once daily from P7 to P34 in rats, and BrdU was administered at P34 after the drug was discontinued (Chen et al., 2009). For neurogenesis studies, the age of the animal, dose of the drug, and time-point at which the BrdU is administered relative to the timing of drug or insult and when the brain is inspected are all key determinants to the results and produce challenges in comparing studies and interpreting results. Fleiss et al. reported sex-dependent effects in the neonatal hypoxic-ischemic-lipopolysaccharide (HI-LPS) model of brain injury. We did not note sex-related differences; this difference likely relates to the older age at which the neurogenesis was assessed in our study (Fleiss et al., 2012).

Interpretation of the cognitive impairments associated with valproate administration is further complicated by the associated toxicity, as evidenced by the increased mortality and impaired weight gain. Toxicity with valproate in rodents has been previously described. Valproate administered to rats, over a range of ages starting at P10 and over a range of doses (which includes the dose we administered) produced a drop in platelets, reduced weight gain, and an increased urine creatinine concentration (Espandiari et al., 2008). While TSA is a selective HDAC inhibitor, valproate has multiple mechanisms of action including gamma-aminobutyric acid (GABA) enhancement, *N*-methyl-D-aspartate (NMDA) receptor modulation, and sodium channel blockade (Monti et al., 2009). It is likely that the other mechanisms of action account for the negative effects of valproate, perhaps in combination with HDAC inhibition. On the other hand, it has been reported that HDAC6 inhibition can be protective in the setting of oxidative stress (Kalin et al., 2012); TSA inhibits HDAC6, but valproate does not. Furthermore, valproate-treatment is associated with increased HDAC2 degradation not seen with TSA-treatment (Kramer et al., 2003). Therefore, it is possible that the differences in HDAC selectivity account for some part of the noted valproate toxicity. It is possible that a lower dose of valproate would have produced the same effect on neurogenesis without the negative effects upon mortality, weight gain and cognitive function; however the measured serum levels suggested that the dose being given was not producing sustained therapeutic (anti-convulsant) levels.

The dose of valproate we chose is reported in the literature to have anticonvulsant and neuroprotective effects, both acutely and chronically (Iyer et al., 1995; Kabakus et al., 2005; Brandt et al., 2006) and peak levels were reasonable and therapeutic based on other rodent studies. However the pharmacokinetic serum levels reported here suggest that, at least in normal juvenile mice chronically administered valproate 200 mg/kg twice daily, the half-life of valproate by P26 is very short and therapeutic drug levels are not being maintained. We previously documented a half-life of 4.5 h with a single dose of 200 mg/kg of valproate in P19 CD1 mice (Markowitz et al., 2010). These data suggest that the half-life of valproate decreases with age between P19 and P26, or that the chronic dosing up-regulates the metabolism of the drug. The mortality associated with the 200 mg/kg twice-a-day dose prevents increasing the dose further to obtain more consistent blood levels, and the immature mice are too small to have a continuous pump inserted. Therefore, it is not currently possible to obtain sustained therapeutic levels of valproate in these animals.

In conclusion, the impact of HDAC inhibitors upon post-stroke neurogenesis is likely to depend on age, duration of treatment before and after BrdU labeling, and timing of both treatment and assessment after the ischemic brain injury. Future studies will need to determine the extent of histone deacetylation and mechanism(s) producing the increased post-stroke neurogenesis, as well as the long-term functional impact of HDAC inhibitor-enhanced neurogenesis. Differences in HDAC selectivity of function could contribute to differences in the other systemic and cognitive effects of valproate compared to TSA and deserve further study. Increasing post-stroke neurogenesis after neonatal stroke is theoretically one approach to enhancing cognitive recovery. With this goal in mind, the data presented here suggests that HDAC inhibition may be one strategy for obtaining increased post-stroke neurogenesis. However, many questions and potential obstacles remain to be addressed before pharmacologic treatment with a selective HDAC inhibitor can be actively promoted for the treatment of neonatal stroke. A stronger link between the increased neurogenesis and improved functional outcome in neonatal ischemia models after treatment with histone deactylase inhibitors needs to be pursued with future studies. Concerns exist as to the impact of TSA upon normal brain development and it may be that a more selective histone deactylase inhibitor would ultimately be a better choice should studies with HDAC inhibitors ever come to clinical trial. Finally, epilepsy after neonatal stroke in babies is a concern and further studies are needed to determine whether HDAC inhibitors impact the risk of developing epilepsy after neonatal stroke and, if so, how.

## Conflict of Interest Statement

The authors declare that the research was conducted in the absence of any commercial or financial relationships that could be construed as a potential conflict of interest.

## Abbreviations

BrdU: bromodeoxyuridine
DG: dentate gyrus
GCL: granule cell layer
HDAC: histone deacetylase
SGZ: subgranular zone
TSA: trichostatin A
VPA: valproate, sodium valproate
